# The high cost of unpaid care by young people:health and economic impacts of providing unpaid care

**DOI:** 10.1186/s12889-020-09166-7

**Published:** 2020-08-05

**Authors:** Nicola Brimblecombe, Martin Knapp, Derek King, Madeleine Stevens, Javiera Cartagena Farias

**Affiliations:** Care Policy and Evaluation Centre, Department of Health Policy, London School of Economics and Political Science, London, UK

**Keywords:** UK, Unpaid/informal care, Long-term care, Young adult, Economic impact, Health, Employment, Inequalities

## Abstract

**Background:**

Many countries worldwide have experienced reductions in provision of formal long-term care services amidst rising need for care. Provision of unpaid care, meanwhile, has grown. This includes care provided by young people. Care responsibilities can affect a young people’s health, education and employment. We aimed to investigate the impacts on the employment and health of young people aged 16 to 25 of providing care, and the associated individual and public expenditure costs.

**Methods:**

We examined employment, earnings and health impacts for individuals, and a range of economic impacts for society, focusing on young people aged 16 to 25 providing unpaid care in England. We applied regression analysis to data from three waves of the UK Household Longitudinal Study (2013/2015, 2014/2016, and 2015/2017) to compare employment and health outcomes among carers and non-carers, and two-part Generalised Linear Models to estimate costs. To address potential selection bias, we then used propensity score matching methods to explore outcomes for a matched sub-sample of young adult carers who started providing care at baseline (2014/16).

**Results:**

Young people aged 16 to 25 who provided care at baseline (2014/16) were less likely to be in employment, had lower earnings from paid employment, and had poorer mental and physical health at follow-up (2015/17) compared to young people of the same age who were not providing care at baseline.. There were substantial costs to the state of young adults providing care from lower tax revenue, welfare benefit payments, and health service use. In aggregate, these costs amounted to £1048 million annually in 2017.

**Conclusions:**

High individual impacts and costs to the state of providing unpaid care, and the potential of such impacts to compound existing inequalities, have many implications for policy and practice in the health, social care, employment and welfare benefits sectors. In particular, the findings reinforce the case for reducing the need for young people to provide unpaid care, for example through better provision of formal care services, and to provide ongoing support for those young people who *do* provide care. As impacts are seen in a number of domains, support needs to be multidimensional.

## Background

Ageing of the population and people living more years with disability mean that provision of long-term care has become an increasingly important societal issue and policy concern internationally [1, 2]. A key component of long-term care provision is unpaid care (sometimes called informal care), and here too there has been increasing attention in policy discussion in some countries. Current policy in England focuses on carer outcomes and needs, in particular employment, but also health, carers’ rights, and support for carers [3]. This attention has included growing concern about *young carers* (defined as aged under 16 or 18 years) and, more recently but with less emphasis, on *young adult carers* (aged 16 to 25). In the UK, important early research on children and young people who provide care was the pioneering and enduringly influential work of Becker and Aldridge [4, 5]. In terms of policy and practice, in England there is now specific provision for young and young adult carers. This includes an assessment of their needs and a transition assessment as they approach age 18 [3, 6].

That there is new provision for a transition assessment for young carers is welcome, although arguably the period could be extended as there are continuing key transitions into adulthood beyond age 18. Between the ages of 16 and 25 – which is the part of the life-course on which our research focuses – individuals experience many important, indeed arguably life-defining transitions such as entering further or higher education, starting employment, and/or leaving home. Because of their caring responsibilities, young adult carers may experience difficulties with many of these important transitions [5, 7]. Leaving the family home, for whatever reason, can be a particular challenge for young adult carers [5].

Previous research on unpaid care identifies socio-demographic inequalities in who provides care and who needs care [8]. Provision of care depends on individual preferences, capacities and circumstances, the latter including family composition such as lone-parent families and family size [9]. There is also an association between provision of care and structural factors such as income, social class, educational level, ethnicity and gender [8]. In many cases, care provided and received by young people and children results simply from lack of alternative sources of support; family structure, low income and poverty may contribute to this situation [9, 10]. Provision of care is also affected by normative expectations about who provides care, gender roles, and norms around reciprocity [11]. For young people there are norms around childhood that may be contravened by taking on ‘adult’ responsibilities and inverting who cares for whom [12]. Caring responsibilities may thus both intersect and conflict with other norms and roles for young people, and may mean taking on multiple, sometimes contradictory, roles.

Providing care may impact on young adults in a number of ways. This includes effects on their employment [13, 14], education [15, 16], and mental and physical health [17]. In England, needs in these areas are explicitly part of the assessment of young adult carers required by the Care Act 2014 and Children and Families Act 2014. In addition, young adult carers experience high levels of individual and household poverty and material deprivation [5].

The research reported here aims to contribute to a better understanding of the impacts of providing care during these transition years by examining those potential impacts longitudinally, and by estimating the costs associated with these impacts. To our knowledge these economic impacts have not previously been estimated. Economic impacts under study include costs for individuals and/or for government.

## Methods

We aimed to answer the question: what are the impacts on the employment and health of young people aged 16 to 25 who provide care, and what are the associated individual and public expenditure costs? Public expenditure costs are costs to government, also referred to as costs to the state. A cost to the state could be a benefit to an individual and vice versa, or could be a cost for both. Using data from the UK Household Longitudinal Study (UKHLS) [18]*,* we compared a range of outcomes at one time-point (2015/2017) for young people who were or were not providing unpaid care at an earlier time-point (2014/2016). These potential outcomes were: being unemployed; leaving employment; physical and mental health; earnings from employment; tax revenue; welfare benefits; and health service use.. We explored the relationships between providing care and consequences for carers firstly by comparing outcomes at time 2 for young people with and without caring responsibilities at time 1, controlling for factors associated with carer’s employment and health. Secondly, to deal with endogeneity issues and using additional data from Wave 5 (2013/2015), we explored the consequences of providing care for new carers (i.e. young people who were carers in 2014/16, but did not have caring responsibilities at the previous wave (2013/2015) by comparing them to non-carers.

### Data and sample

The UKHLS started in 2009, and collects data annually from a sample of household members aged 10 or older living in the UK. It built on, and incorporated study members from, the British Household Panel Survey (BHPS). Sampling is based on a proportionately stratified, clustered sample of addresses selected by postcode, supplemented by specific additional samples added at subsequent waves [19]. Wave 7 offers the most recent data available, with an overall sample of 42,217. This comprised members of: the BHPS and Northern Ireland Household Panel Survey, the ‘general population sample’ and the ‘ethnic minority boost sample’, the latter two both originally sampled at Wave 1 of the UKHLS. For the first set of analyses, our sample comprised all panel members who took part in the study in both Wave 6 (2014/2016; hereafter called time 1) and Wave 7 (2015/2017; hereafter time 2), who were aged 16 to 25 in Wave 6, and for whom data about caring responsibilities were available (*n*= 6866: 561 carers; 6342 non-carers). The second set of analyses included only those individuals who were *not* providing care in the previous wave (i.e. Wave 5, 2013/2015, hereafter called time 0). This wave (with overall sample of 4067), included 254 new carers and 3813 non-carers, and so includes panel members who took part in the study at all three waves. Ethical approval for the UKHLS was obtained by the University of Essex Ethics Committee which has approved all data collection on Understanding Society main study and innovation panel waves, including asking consent for all data linkages except to health records.

### Measures

#### Caring responsibilities

The variable for caring responsibilities was derived from two questions asked of respondents at time 1: ‘Is there anyone living with you who is sick, disabled or elderly whom you look after or give special help to (for example, a sick, disabled or elderly relative/husband/wife/friend etc.)?’ and ‘Do you provide some regular service or help for any sick, disabled or elderly person not living with you?’.

Our sample comprised respondents who answered yes to either of these questions. We included young people caring for a person with care needs of any age. In line with the 2011 Population Census definition, we excluded sample members caring for a ‘client of a voluntary organisation’ only.

#### Employment and health

We considered four employment and health outcomes at time 2: employment status; leaving employment; physical health; and mental health. Employment status was recoded into two categories: 0 = in paid- or self-employment; 1 = unemployed or not working due to being long-term sick or disabled. The ‘leaving employment’ variable also had two categories: 0 = in paid- or self-employment at both times; 1 = in paid- or self-employment at time 1, left employment by time 2. People who were not employed at time 1 were therefore excluded from this particular analysis, although included in other analyses. For health outcomes, the variables used were the Physical and Mental Component Scores of the Short-Form 12 Health Survey (SF12, PCS and MCS) which measure physical and mental health respectively; they have been validated for use in the general population [20]. Lower scores indicate poorer physical/mental health.

#### Individual and public expenditure costs

We estimated a range of individual and/or public expenditure costs at time 2 associated with health, employment, and potentially other impacts on carers. For example, lost tax revenue is a cost associated with unemployment. Health service use will often follow from poor health and generates costs, although the association may not be straightforward: for example, people in this age group under-use health services for mental health needs [21, 22]. Welfare benefit receipt is associated with unemployment, low-paid employment, caring responsibilities in conjunction with low or no income from employment, or ill-health/disability.

The four costs we estimated were differences between carers and non-carers in monthly earnings, annual tax revenue, monthly welfare benefits, and health service use. The variable for monthly earnings was based on a question about earnings from paid employment and thus excluded earnings from self-employment. We also excluded sample members who were in full- or part-time education or training at the same time as paid employment. Monthly welfare benefit income was based on a question asking about other sources of income for the individual. Tax revenue was calculated by deducting net earnings from gross earnings to give tax paid. This will include income tax, national insurance contributions, and any other deductions such as any pension contributions (although the latter will be rare for this age group). We again excluded people who were simultaneously in full- or part-time education or training.

Health service costs were calculated from data on GP visits, outpatient visits, and inpatient stays. (No other healthcare contact questions were asked in the survey.) For GP and outpatient visits, questions asked about visits in the previous year; possible responses were ‘none’, ‘one or two’, ‘three to five’, ‘six to ten’ or ‘more than ten’. Apart from ‘more than ten’, where we took the low point (i.e. 11), we took the mid-point of each range and multiplied it by the relevant UK unit cost [23]. Inpatient costs were estimated from number of hospital inpatient days in the previous year reported by the respondent at time 2, multiplied by the NHS 2016/17 elective and non-elective combined excess bed day cost [24].

### Analysis

Using the whole sample of young people aged 16 to 25 for whom we had information on caring responsibilities at time 1, we classified young people into two groups: those with and without caring responsibility at time 1. We then compared their mean values or frequencies for socio-demographic characteristics at time 1 (baseline) and the two groups’ employment, health and economic outcomes at time 2. Chi-squared tests and tests of means (t-test) were used to determine any statistically significant group differences.

For further comparison of outcomes between those with and without caring responsibilities we carried out multivariate analyses: logistic regression models for categorical variables and Generalised Linear Models (GLM) for continuous variables. The models controlled for covariates potentially associated with carers’ employment and health, based on previous research (e.g. [9, 25]) and our initial bivariate analyses for this sample. For employment status, leaving employment, earnings from paid employment, state welfare benefits, and forgone tax revenue, the covariates we examined were: sex of carer (male = 0; female = 1); carer’s ethnicity (White = 0; Black, Asian and Minority Ethnic groups (BAME) = 1); carer’s health at time 1 (SF12 MCS and PCS scores time 1); carer’s marital status (single = ‘0’; married/living with partner/in civil partnership = ‘1’); carer’s highest educational qualification (degree or higher degree, A-level, GCSE, none); carer’s age; and housing tenure (owner-occupied, private-rented; social-rented). Social-rented housing (or ‘public housing’) in the UK is provided at more affordable rents, usually by local government or (non-profit sector) housing associations. Reduction of the amount of social housing in the UK and tighter eligibility criteria have led to a situation where social housing is increasingly likely to be occupied by households with low employment, low income and/or low wealth.

For mental and physical health scores and health service costs, the covariates were: carer’s sex, ethnicity, marital status, highest educational qualification, carer’s age, and housing tenure. Other possible covariates were excluded because of multicollinearity.

For cost analyses, we used two-part Generalised Linear Models [26]. As the dependent variables may have skewed distributions with a substantial number of zeros, we used a modified Park test [27] to select the appropriate distribution and link function. The marginal effect of providing care at time 1 on each cost measure at time 2 could then be estimated from each regression model: this represents the mean cost at time 2 associated with a young person (aged 16 to 25) providing care at time 1 compared to an equivalent young person *not* providing care at time 1. An advantage of this modelling is that it uses the whole sample of young people for whom we have information about care responsibilities. This not only means the sample size is maximised, but, and this is particularly important for this age group, includes people who have been caring for a long duration, including in childhood.

However, a challenge when examining relationships between caregiving and health and employment outcomes using observational data is a fundamental identification problem that can lead to biased estimates of the causal effect of unpaid care on these outcomes (e.g. [28]). For example, people with poorer employment prospects may be more likely to ‘select into’ providing unpaid care and also to have worse labour market outcomes [29, 30]. (We should note, however, that there are dissimilarities for children and young people in routes into providing care compared to the older aged carers who have been the subject of previous research [31, 32]). We sought to address possible selection bias in a number of ways. First, we performed regression models, described above, that considered care provision at time 1 and outcomes at time 2, controlling for a number of factors suggested from previous research as likely to be associated with providing care and/or associated with the outcomes under study - [9, 25, 30]. Cost estimates were based on two-part models which have been shown in previous research to be robust to endogenous selection (see e.g. - [33]).

Additional ways to address potential bias were also investigated. We considered three approaches: fixed effects (FE), instrumental variables (IV) and propensity score matching (PSM). FE can be used to address potential endogeneity when the endogenous variable is constant over time. However, it does not allow for analysis of the effect of variables that are not constant over time and results in a very much reduced sample size, thus undermining one of the advantages of this dataset. In addition, FE analysis was not possible to perform when looking at the cost measures because data on costs were only available at one wave. IV analysis, sometimes also used to deal with selection bias, uses the full sample but faces the challenge of identifying a truly exogenous instrumental variable. (Instrumental variables should be correlated with care provision but expected to be uncorrelated with the outcome of interest.). Furthermore, IV analysis is not suitable for use with an outcome variable with a large number of zero values, as is the case with our cost data. Another alternative was to perform a Heckman correction model which adjusts for self-selection [34], but, as with IV methodology, it requires a robust exogenous component to obtain unbiased estimations [35].

Propensity Score Matching, on the other hand, allowed us to take advantage of a fuller sample and to use two-part models to estimate costs. This methodology is based on the estimation of a propensity score which refers to the probability of treatment or participation (in our study, the probability of providing unpaid care), conditional on observed baseline characteristics [36]. The PSM approach aims to identify a group of non-carers (counterfactual/comparison group) who are most similar to carers across a spectrum of observable features at baseline (before care provision started), to then compare their outcomes at a later time-point.

PSM requires that individuals are matched prior to treatment and our analyses therefore were based on a sub-sample which included only those young people who were non-carers at the wave prior to time 1, i.e. time 0 (wave 5; 2013/2015). By time 1, some had become carers and some had not. Those who became carers (new carers; the so-called ‘treated’ sample in the PSM literature) were matched to those who did not (non-carers; ‘untreated’ or comparator sample). ‘Treatment’ was thus defined as acquiring caring responsibilities between 2013/2015 and 2014/2016 (time 1). Matching variables were based on factors demonstrated in previous research to influence both the participation decision (being a carer) and the outcome variable [37]. We used matching variables which were either constant over time or measured before participation, i.e. before starting providing care at time 0 [37]. Matching variables varied by outcome in our analysis but include carer’s gender, ethnicity, highest educational qualification, health, marital status, and housing tenure in 2013/2015. All outcomes were measured in the subsequent wave (2015/2017). We calculated the average treatment effect (ATE): the difference in outcomes between the two groups (receiving ‘treatment’ compared to not receiving ‘treatment’). Bootstrapping was used to estimate standard errors and construct confidence intervals of the estimated treatment effect. Kernel matching was performed in order to achieve a lower variance while making use of a weighted average of all non-carer individuals to be part of the counterfactual group. Tests were carried out to assess the balance of the final models. We report two measures of covariate balance post-matching: Rubin’s B (the absolute standardised difference of the means of the linear index of the propensity score in the treated and matched non-treated group), and Rubin’s R (the ratio of treated to (matched) non-treated variances of the propensity score index). If the value of B is less than 25 and if R has a value between 0.5 and 2, this is interpreted as indicating that the samples are sufficiently balanced [38].

A potential disadvantage of PSM is that it includes ‘new’ carers only. As stated elsewhere, many young adult carers have been caring since childhood, and so would not be included in the PSM sample. We therefore report findings from both the whole young adult carer sample (unmatched) models and from the new carer-only sample (matched) models, and comment on any differences between them.

To estimate annual aggregate costs to the state of young people providing care we used (i) mean cost differences for all carers; (ii) post-matched average treatment effects for new carers (treatment being the acquisition of caring responsibilities between 2013/2015 and 2014/2016). We multiplied per-person mean differences in annual lost tax revenue, annual welfare benefits (monthly welfare benefits multiplied by 12) and annual health service costs by the estimated numbers of people aged 16 to 25 with caring responsibilities. For the estimates for all young adult carers we used the prevalence of being a young adult carer in *Understanding Society* (8.1%) and the estimated numbers of young people aged 16–25 in mid-2015 [39]. For the aggregate estimates for new carers, we used prevalence of acquiring new caring responsibilities between 2013 and 2015 in *Understanding Society* (5.7%).

All tests of statistical significance used robust standard errors. A significance level of 0.05 was used as the criterion to determine statistical significance and 0.10 to determine marginal significance. We conducted analyses using Stata 14.2 [40].

## Results

The results section is laid out as follows. Table 1 presents descriptive statistics of the complete sample of young people aged 16 to 25 for whom we have information about caring responsibilities at baseline and the outcomes under study. Table 2 reports firstly the results of the logistic and GLM regression models for all carers; secondly, for new carers only, the difference in outcomes between the two matched groups (average treatment effect; ATE). The final column in Table 2 shows the results of tests to assess the balance of the final models: Rubin’s B and Rubin’s R.

Table 1 shows that, compared to non-carers of the same age, young adult carers in our sample were more likely to be female, from white ethnic background, de facto married, have lower educational qualifications, and live in social housing. A higher proportion of young people aged 16 to 25 who had caring responsibilities at time 1 were unemployed or economically inactive due to long-term sickness or disability at time 2, compared to young people without caring responsibilities. Young adult carers had worse physical and mental health at time 2 compared to non-carers. Mean monthly earnings from paid employment were significantly lower for carers than non-carers; monthly welfare benefits, forgone tax revenue, and health service costs were significantly higher. It is important not to over-interpret these descriptive summary findings as we have yet to take make any adjustments for the range of potential influences of being a carer.

**Table 1:** Sample characteristics, outcomes, and mean costs for young people aged 16–25 with and without caring responsibilities: all carers

CARER CHARACTERISTICS TIME 1	Aged 16–25, not providing unpaid care time 1 (***N*** = 6342; 91.9%) %	Aged 16–25, providing unpaid care time 1 (***N*** = 561; 8.1%) %
Female*	50.4	58.3
Ethnicity*		
White ethnicity	69.4	73.8
Black, Asian and minority ethnic	30.6	26.3
Physical health score*	54.8	53.3
Mental health score*	48.2	46.7
Married, living with partner, in civil partnership*	13.2	16.5
Highest educational qualification*		
Degree or higher degree	33.1	25.2
A-level	38.9	25.2
GCSE	24.6	30.0
None	3.5	4.1
Housing tenure*		
Owner-occupied	56.4	52.0
Private-rented	22.3	14.9
Social-rented	21.3	33.2
**EMPLOYMENT AND HEALTH TIME 2**		
Unemployed or long-term sick/disabled*	16.1	24.4
Left employment*	4.6	9.9
Physical health score*	54.6	53.2
Mental health score*	47.6	45.3
**COSTS TIME 2**	**Mean (£)**	**Mean (£)**
Monthly net earnings from paid employment*	870	647
Annual tax revenue*	2096	1324
Monthly individual state welfare benefits*	81	150
Annual health service use*	310	686

*p < .05

Physical health score is Physical Component of the Short-Form 12 Health Survey (SF12 PCS); lower score = worse physical health. Mental health score is Mental Component of the Short-Form 12 Health Survey (SF12 MCS); lower score = worse mental health

Table 2 presents the results of the regression models of the consequences at time 2 associated with being a carer at time 1. Column 2 shows the results for all carers (unmatched sample). Controlling for covariates, young adult carers had higher odds ratios (OR) of being unemployed or not in employment/ long-term sick or disabled at time 2 compared to non-carers (OR = 2.39; 95% CI = 1.60, 3.56). They were more than twice as likely to have left employment and become unemployed (OR = 2.52; 95% CI = 1.34, 4.73). They had worse physical and mental health than non-carers.

Mean cost differences at time 2 between carers and non-carers at time 1 are also shown in Table 2. We looked at employment-related costs in a number of ways, in each case controlling for covariates. Looking at all young adult carers at baseline (Table 2, column 2), net earnings from paid employment were on average £164 a month lower than those of non-carers. This is an individual cost to the young person and (if living with others) to the household. This is, in part, mitigated by welfare benefits. However, even accounting for income from this source, young people who provide care were still around £100 less well-off per month compared to non-carers. In terms of state costs, tax revenue was £741 a year lower and welfare benefit costs were £44 higher a month (or £528 a year). Health service costs were £289 a year higher.

**Table 2:** Regression analyses of associations between providing unpaid care at time 1 and economic and health outcomes at time 2

OUTCOMES AT TIME 2	Caring responsibilities time 1 compared to no caring responsibilities at time 1: all carers^**1**^	Average treatment effect (ATE) after propensity score matching: new caring responsibilities at time 1 compared to no caring responsibilities at time 1	PSM diagnostic tests
	Odds ratio (95% CI)	ATE (95% CI)	
Unemployed/long-term sick or disabled	2.39* (1.60, 3.56)	0.17* (0.07, 0.26)	B: 20.9
			R: 0.60
Left employment	2.52* (1.34, 4.73)	0.04 ns (−0.04, 0.11)	B: 12.5
			R: 1.05
	**Coefficient (95% CI)**		
Mental health score	−2.75* (−4.32, −1.17)	−1.47 ns (−3.77, 0.83)	B: 23.3
			R: 0.63
Physical health score	−1.00 ~ (−2.08, 0.08)	−2.03*. (−3.79, −0.27)	B: 23.3
			R: 0.63
	**Mean cost difference (£) (95% CI)**		
Monthly earnings from employment	−164.53* (− 264.28, −64.78)	− 225.65* (− 351.48, −99.83)	B: 21.1
			R: 0.67
Monthly individual state welfare benefits	44.27* (10.05, 78.49)	110.83* (32.78, 188.89)	B: 21.1
			R: 0.67
Annual tax revenue	− 740.81* (− 1268.84, − 212.78)	− 644.25 ~ (− 1359.64, 31.27)	B: 21.1
			R: 0.67
Annual health service use costs	289.01* (111.18, 466.85)	707.60* (68.85, 1346.35)	B: 22.6
			R: 0.62

*p < .05; ~ *p* = 0.10

B = Rubins’ B (the absolute standardised difference of the means of the linear index of the propensity score in the treated and matched non-treated group), R = Rubin’s R (the ratio of treated to (matched) non-treated variances of the propensity score index). If B < 25 and R = 0.5 to 2, the samples are considered sufficiently balanced

[1] Controlling for carer’s sex, ethnicity, health, marital status, highest qualification, housing tenure and age at time 1 in analysis of employment status; controlling for carer’s sex, ethnicity, health, marital status, highest qualification, housing tenure and age in analysis of earnings from paid employment, lost tax revenue, and welfare benefits; controlling for carer’s sex, ethnicity, marital status, highest qualification, housing tenure at time 1 in analysis of physical health score; mental health score; controlling for carer’s sex, ethnicity, marital status, highest qualification, housing tenure and age in analysis of health service use. Physical health score is Physical Component of the Short-Form 12 Health Survey (SF12 PCS); lower score = worse physical health. Mental health score is Mental Component of the Short-Form 12 Health Survey (SF12 MCS); lower score = worse mental health

The full logistic and GLM regressions (Additional file 1: Table 3) show that odds of not being in employment at time 2 were significantly lower for women compared to men, higher for young people from BAME backgrounds compared to white ethnic backgrounds, and for young people with lower educational qualifications, poorer physical and mental health, or living in social-rented housing compared to owner-occupied housing. Young people with lower educational qualifications had poorer physical, but not mental, health. Individuals living in social-rented accommodation had poorer mental and physical health compared to those in owner-occupied accommodation. Lost tax revenue was higher for individuals with poorer health, with lower qualifications, or living in social-renting households (Additional file 2: Table 4). Earnings were lower and welfare benefits higher for women, those with poorer health, and those with lower qualifications. Women reported worse mental health than men (Additional file 1: Table 3) and their health service use costs were higher (Additional file 2: Table 4). Although individuals living in social-rented accommodation had poorer health compared to those in owner-occupied accommodation, accommodation type was not related to health service costs.

After propensity score matching, comparing ‘new’ carers to matched non-carers, those providing care were still less likely to be in employment and to have poorer physical health than their matched counterparts (Table 2, column 3). The differences between the two groups in likelihood of leaving employment and mental health were not statistically significant after matching. Earnings from paid employment and tax revenue were significantly lower for individuals newly providing care compared to matched non-carers. Welfare benefits and health service costs were all significantly higher.

Based on prevalence of all young people aged 16 to 25 providing unpaid care from our *Understanding Society* sample, aggregate *public expenditure* costs - costs to the state - of young adults providing care were £1048 million annually, comprising £497 million in forgone tax revenue; £357 million for welfare benefits; and £194 million for health service costs. For ‘new’ carers, after matching with non-carers with similar characteristics at baseline, aggregate *public expenditure* costs were £1256 million annually. Excluding tax revenue, which was statistically significant at the *p* = 0.10 level (*p* = 0.061), this total was still £954 million a year.

## Discussion

In our study, young people aged 16 to 25 who provided care were found – one year later – to be subsequently less likely to be in employment and to have poorer health than equivalent young people who did not provide care. These findings are consistent with previous cross-sectional research [5, 13, 16, 17]. This is the case both for all carers and, with the exception of mental health, for new carers matched with their non-caring counterparts on a number of baseline characteristics to control for selection into unpaid care. The difference in findings in relation to mental health between the matched and unmatched samples may be because of differences between short- and long-term effects of unpaid care: the finding from the unmatched analysis may be reflecting an association between poorer mental health and longer duration of care provision. However, this difference may instead be due to the reduced sample size used in the matched analysis.

We also found sizeable associated costs to individual and society, and again this is the case both for all carers and for new carers matched with their non-caring counterparts. Compared to non-carers, young adult carers at time 1 had lower earnings from paid employment at time 2. Even taking into account welfare benefits received, this represents an impact on both individual and household income. We also found substantial costs to the state in terms of lost tax revenue, welfare benefits, and health service use. To the best of our knowledge, our study is the first to estimate these costs, and also the first to explore these associations using longitudinal data.

The reasons for negative employment-related consequences for young adult carers are in some respects similar to those previously observed for other unpaid carers, although there are some differences. Providing care can be incompatible with entering or maintaining employment [13, 41, 42], which will generate costs to the state in terms of welfare benefits and forgone tax revenue. Carers who do enter or stay in paid employment tend to work fewer hours than non-carers [43], which will also impact on earnings and tax revenue [44].

Many young adult carers begin caring at a very young age, often whilst at school [5, 45], which means they have already been caring for a significant period by the time they reach a point of wishing to enter employment. The responsibilities of being a carer at such a critical life-stage for educational development and attainment can have life-long consequences. Young carers may experience difficulties at school with, for example, attendance, disruption and completing homework [46]. Cross-sectional studies show an association between being a young carer and lower educational attainment at GCSE-level [15], and a negative association between caring and going on to further or higher education [47, 48]. Of those who do continue into further or higher education, many experience difficulties combining caring responsibilities with academic commitments, and there are high drop-out rates [15].

Lower educational qualifications impact on ability to enter employment and earnings [49]. In our study, effects of caring were seen even after taking into account educational qualifications, suggesting that caring itself impacts on employment. However, young people with lower qualifications were less likely to be in employment and more likely to have left employment. There can also be an expectation that young carers will continue to provide unpaid care rather than enter employment [14]; having lower educational qualifications may contribute to this expectation, and may limit the other alternatives available. The length of time a young person has been providing care and the age at which they start may contribute to the normalisation of the caring role and to expectations that they will continue in that role [31].

Carers (in general) have been found in previous research to have poorer mental and physical health than non-carers [43], and young adult carers are no exception [17]. As we found in this study, the difference is particularly marked with regard to mental health. Providing care, especially at high intensity, is associated with stress, anxiety, depression, loneliness, and isolation for carers in general [2]. For young adult carers the risks might be even higher: care is being provided during critical periods for their mental health as well as for their education and employment, given that the peak age of onset in the general population for most common mental disorders is 8 to 15 years and three-quarters of adulthood mental health problems start by the age of 18 [50, 51].

Poorer health is associated with higher health service costs. As we commented earlier, under-use of health services for mental health needs in this age group – which occurs for various reasons – means that the relationship is not completely straightforward. A carer’s poorer health may also impact on their employment prospects. We found effects of caring on employment even after taking carer’s health into account. However, poorer health was associated with a higher likelihood of being unemployed and leaving employment.

In estimating the costs associated with providing unpaid care, we are not necessarily implying that these should be seen as negative. Clearly it is important that young adult carers receive the welfare benefits they need and to which they are entitled, and also that they access the health services they need. Nonetheless these are high costs to government – totalling £1048 million annually. This money might be better spent on preventing negative consequences, for example by reducing or removing the need for young people to provide care through, for example, better provision of services for the person they care for, which could additionally benefit the care-recipient. It could also be achieved through initiatives to support the education, training, employment, financial situation, and physical and mental health of young adult carers. For example, appropriate support and understanding in educational settings can make a difference to a young adult carers’ engagement and achievement [5]. Support in workplaces has been found valuable by working-age carers more generally [52, 53]. Further examples include addressing the pervasive under-provision of young people’s mental health services [54] and of youth services more generally [55].

Unpaid care by young people is not provided, received or needed equally across society, nor does it impact on everyone equally, either economically or otherwise. Disadvantage and social exclusion reduce choices about who provides care and so create tensions between preferences for how care needs are met and the ability to meet those preferences [10]. That these carers then experience negative economic and other consequences is further compounding this disadvantage.

There are a number of limitations to our study. Firstly, there are difficulties with identifying young adult carers both in surveys and more generally. *Understanding Society* avoids some of the difficulties by asking the young people themselves about care provision and by avoiding the term ‘carer’ (instead using phrases such as ‘look after or give special help to’). Nonetheless, our sample selection may have missed some young adult carers, and the prevalence we use may therefore be an underestimate, particularly because the question does not specify giving help to someone with mental ill-health or drug or alcohol dependency, and these are needs that are supported by young carers more than other carers [56]. Another potential issue may be the degree of fluidity in caring responsibilities seen in both this dataset and for carers more generally. This means that for some young adult carers, caring responsibilities may have changed over the period of our study.

With regard to the measurement of consequences, there is risk of recall bias, in particular in relation to service use, although self-report is considered an acceptably accurate method for collecting service use data [57], and *Understanding Society* asks about service use over the last year only. The measure of self-reported earnings was based on information about the previous month, which is a short timeframe, and only includes earnings from paid employment, thereby excluding people in self-employment. However, very few people at this age are self-employed: only 2.3% of the sample for whom we have employment status data. Our measure of tax revenue is based on earnings from paid employment, and so excludes people in self-employment. Tax revenue was calculated as the difference between gross and net earnings and so may include other deductions such as any pension contributions. This measure may therefore overestimate tax paid. However, pension contributions are likely to be a very small proportion of deductions, especially for this age group (for example, auto-enrolment into pension schemes in England is only for people aged 22 and above). Although we estimated a number of costs, there may be other economic impacts of providing care, such as school absenteeism and its impact on school and other staff. The relationship between care provision and economic impacts can be subject to endogeneity or selection bias, with a resultant over- or under-estimation of the economic impacts of being a carer. However, we observed substantial economic impacts even after using analysis techniques which address these possible biases.

Our study has strengths, in particular that it is based on analysis of longitudinal data for a large nationally representative sample. This means, for example, that we had sufficient data on provision of care by young adults at one time-point and on a wide range of consequences 1 year later. Longitudinal exploration of this kind is very rare, and some of the economic consequences that we looked at have not previously been studied in this way.

We looked at a range of short-term impacts of young people (aged 15–24) providing unpaid care. There are likely also to be long-term consequences, including economic impacts. Being unemployed at a young age impacts on the young person’s life-long economic outcomes: young people are more affected than other age groups by long-term unemployment [58]. This will generate long-term costs to individuals and the state. Having low or no income from paid employment over time impacts on the accumulation of wealth over the life-course. For health, the majority of mental illnesses that start during adolescence persist into adulthood [50, 51], again with associated costs.

## Conclusion

We found high economic costs to individuals and the state as a result of young people providing unpaid care, the latter amounting to an estimated £1 billion a year. For young adults, provision of unpaid care often takes place over a long duration and during critical periods for their education, mental health, and transitions into adult life. By estimating the economic impacts to both individuals and society, our study adds to an understanding of the consequences of care provision by young adults. Our estimates of the economic impacts of young adults providing care should be taken seriously by policy-makers. This is not only because there are high financial costs of caring to government, but primarily because, for the young people themselves, there can be considerable negative consequences, many of which have strong potential to persist into the long term. In conjunction with inequalities in caring responsibilities across socioeconomic groups and constrained choices for care provision faced by many young people and their families, this evidence on negative consequences reinforces the case for prevention, early intervention, and ongoing support. As impacts are seen in a number of domains, such policy actions need to be multidimensional.

## Supplementary information

**Additional file 1: Table 3.** Regression analyses of associations between providing unpaid care at time 1 and employment status and health outcomes at time 2. Results of the regression models of the consequences at time 2 associated with being a carer at time 1: results for all carers. Data: Wave 6 (2014/2016) and Wave 7 (2015/2017) of the UK Household Longitudinal Study.

**Additional file 2: Table 4.** Regression analyses of associations between providing unpaid care at time 1 and costs associated with economic and health outcomes at time 2. Results of the regression models of the individual and state economic consequences time 2 associated with being a carer at time 1: results for all carers. Data: Wave 6 (2014/2016) and Wave 7 (2015/2017) of the UK Household Longitudinal Study.

## Data Availability

The dataset supporting the conclusions of this article is available under certain conditions in the UK Data Service repository, SN: 6614, *Understanding Society: Waves 1–8, 2009–2017 and Harmonised BHPS: Waves 1–18, 1991–2009*. 10.5255/UKDA-SN-6614-12

## Abbreviations

ATE: Average treatment effect
BAME: Black, Asian and Minority Ethnic
BHPS: British Household Panel Survey
FE: Fixed effects
GLM: Generalised Linear Models
MCS: Mental Component Score of the Short-Form 12 Health Survey
OR: Odds ratios
PCS: Physical Component Score of the Short-Form 12 Health Survey
PSM: Propensity score matching
SF12: Short-Form 12 Health Survey
UKHLS: UK Household Longitudinal Study
