# Supplementary material for: The high cost of unpaid care by young people:health and economic impacts of providing unpaid care
Source: BMC Public Health. 2020 Aug 5;20:1115. doi: 10.1186/s12889-020-09166-7 (PMC7409476; doi:10.1186/s12889-020-09166-7)
Supplement: Supplementary file 1 — Additional file 1: Table 3. Regression analyses of associations between providing unpaid care at time 1 and employment status and health outcomes at time 2. Results of the regression models of the consequences at time 2 associated with being a carer at time 1: results for all carers. Data: Wave 6 (2014/2016) and Wave 7 (2015/2017) of the UK Household Longitudinal Study. [file 12889_2020_9166_MOESM1_ESM.docx]

Additional file 1: Table 3: Regression analyses of associations between providing unpaid care at time 1 and employment status and health outcomes at time 2

|  | **Unemployed/long-term sick or disabled at time 2** | **Left employment time 2** | **Mental health score time 2** | **Physical health score time 2** |
| --- | --- | --- | --- | --- |
|  | **Odds ratio (95% CI)** | **Odds ratio (95% CI)** | **Coefficient (95% CI)** | **Coefficient (95% CI)** |
| Caring responsibilities time 1 compared to no caring responsibilities | 2.39*  (1.61, 3.55) | 2.52*  (1.34, 4.73) | -2.75*  (-4.32, -0.43) | -1.00~  (-2.08, 0.08) |
| Female compared to male | 0.67*  (0.48, 0.93) | 0.78  (0.44, 1.38) | -1.34*  (-2.26, -0.43) | -0.80*  (-1.38, -0.21) |
| Black and minority ethnic compared to white ethnic | 1.51~  (0.99, 2.31) | 1.87~  (0.93, 3.74) | -0.42  (-1.56, 0.71) | -1.76*  (-2.53, -0.99) |
| Mental health score time 1 | 0.95*  (0.94, 0.97) | 0.96*  (0.93, 0.98) | - |  |
| Physical health score time 1 | 0.95*  (0.94, 0.97) | 0.96*  (0.93, 0.99) | - |  |
| Highest educational qualification time 1 (compared to degree/higher degree) |  |  |  |  |
| None | 8.68*  (3.91, 19.28) | ^ | -3.33*  (-6.06, -0.60) | -3.71*  (-5.91, -1.52) |
| GCSE | 3.10*  (2.01, 4.79) | 1.67  (0.83, 3.37) | -1.51*  (-2.82, -0.20) | -2.24*  (-3.16, -1.32) |
| A-level | 1.13  (0.73, 1.76) | 0.63  (0.31, 1.28) | -0.31  (-1.41, 0.79) | -1.09*  (-1.78, -0.40) |
| Married, living with partner, in civil partnership compared to single | 0.82  (0.53, 1.26) | 1.19  (0.57, 2.51) | 0.51  (-0.66, 1.68) | -0.96*  (-1.78, -0.13) |
| Housing tenure (compared to owner-occupied) |  |  |  |  |
| Social-rented | 1.71*  (1.20, 2.44) | 0.90  (0.47, 1.73) | -1.03  (-2.26, 0.21) | -1.92*  (-2.74, -1.10) |
| Private rented | 1.16  (0.75, 1.78) | 0.47~  (0.20, 1.11) | -1.48*  (-2.65, -0.32) | -0.63  (-1.42, 0.16) |
| Age time 1 | 0.90*  (0.84, 0.97) | 0.89~  (0.78, 1.01) | -0.07  (-0.28, 0.15) | -0.00  (-0.14, 0.13) |

*p < .05; ~ p=0.10

Physical health score is Physical Component of the Short-Form 12 Health Survey (SF12 PCS); lower score = worse physical health. Mental health score is Mental Component of the Short-Form 12 Health Survey (SF12 MCS); lower score = worse mental health.
