# Supplementary material for: The high cost of unpaid care by young people:health and economic impacts of providing unpaid care
Source: BMC Public Health. 2020 Aug 5;20:1115. doi: 10.1186/s12889-020-09166-7 (PMC7409476; doi:10.1186/s12889-020-09166-7)
Supplement: Supplementary file 2 — Additional file 2: Table 4. Regression analyses of associations between providing unpaid care at time 1 and costs associated with economic and health outcomes at time 2. Results of the regression models of the individual and state economic consequences time 2 associated with being a carer at time 1: results for all carers. Data: Wave 6 (2014/2016) and Wave 7 (2015/2017) of the UK Household Longitudinal Study. [file 12889_2020_9166_MOESM2_ESM.docx]

Additional file 2: Table 4. Regression analyses of associations between providing unpaid care at time 1 and costs associated with economic and health outcomes at time 2

|  | **Monthly net earnings from employment time 2** | **Annual tax revenue time 2** | **Monthly state individual welfare benefits time 2** | **Annual health service costs time 2** |
| --- | --- | --- | --- | --- |
|  | **Mean cost difference (£) (95% CI)** | | | |
| Caring responsibilities time 1 compared to no caring responsibilities | -164.53*  (-264.28, -64.78) | -740.81*  (-1268.84, -212.78) | 44.27*  (10.05, 78.49) | 289.01*  (111.18, 466.85) |
| Female compared to male | -181.75*  (-253.44, -110.06) | -907.89*  (-1306.91, -508.88) | 109.00*  (80.15, 137.86) | 215.87*  (110.30, 321.45) |
| Black and minority ethnic compared to white ethnic | 13.59  (-118.21, 145.38) | -284.77  (-794.11, 224.57) | -5.83  (-39.15, 27.49) | -13.55  (-129.17, 102.06) |
| Mental health score time 1 | 10.06*  (6.14, 13.98) | 22.22*  (6.65, 37.80) | -2.96*  (-4.07, -1.85) | - |
| Physical health score time 1 | 13.36*  (8.55 18.17) | 26.60~  (-0.55, 53.74) | -2.65*  (-4.06, -1.24) | - |
| Highest educational qualification time 1 (compared to degree/higher degree) |  |  |  |  |
| None | -720.44*  (-858.55, -582.32) | -2005.44*  (-2920.06, -1090.82) | 226.39*  (136.33, 316.44) | 431.25  (-187.52, 1050.02) |
| GCSE | -337.25*  (-429.90, -244.60) | -1500.54*  (-1887.99, -1113.09) | 138.76*  (103.79, 173.73) | 227.33*  (56.40, 398.25) |
| A-level | -114.50*  (-207.89, -21.11) | -538.43  (-1267.58, 190.72) | 57.08*  (28.54, 85.62) | 7.71  (-102.30, 117.72) |
| Married, living with partner, in civil partnership compared to single | -40.78  (-110.46, 28.90) | -234.12  (-777.43, 309.18) | 29.25*  (1.74, 56.77) | 235.34*  (61.51, 409.18) |
| Housing tenure (compared to owner-occupied) |  |  |  |  |
| Social-rented | -192.88*  (-301.71, -84.05) | -1112.19*  (-1611.70, -612.67) | 160.37*  (122.08, 198.66) | 58.54  (-69.47, 186.56) |
| Private rented | -31.91  (-129.82, 66.01) | -429.03*  (-830.1, -27.89) | 73.82*  (42.87, 104.76) | 91.73  (-74.04, 257.51) |
| Age time 1 | 51.90*  (33.88, 69.91) | 450.75  (241.52, 659.98) | 17.31*  (11.45, 23.17) | -13.60  (-37.86, 10.66) |

*p < .05

Physical health score is Physical Component of the Short-Form 12 Health Survey (SF12 PCS); lower score = worse physical health. Mental health score is Mental Component of the Short-Form 12 Health Survey (SF12 MCS); lower score = worse mental health.
